# Coinfections with Respiratory Pathogens among COVID-19 Patients in Korea

**DOI:** 10.1155/2021/6651045

**Published:** 2021-05-12

**Authors:** Kyoung Ho Roh, Yu Kyung Kim, Shin-Woo Kim, Eun-Rim Kang, Yong-Jin Yang, Sun-Kyung Jung, Sun-Hwa Lee, Nackmoon Sung

**Affiliations:** ^1^Clinical Research Institute/Molecular Diagnosis Center, Seegene Medical Foundation, Seoul 04805, Republic of Korea; ^2^Department of Clinical Pathology, School of Medicine, Kyungpook National University Hospital, Daegu 41944, Republic of Korea; ^3^Division of Infectious Disease, Department of Internal Medicine, Kyungpook National University Hospital, Daegu 41944, Republic of Korea

## Abstract

The detection of severe acute respiratory syndrome coronavirus 2 (SARS-CoV-2) in upper and lower respiratory specimens and coinfection with other respiratory pathogens in patients with coronavirus disease 2019 (COVID-19) was investigated. Study subjects (*N* = 342) were retrospectively enrolled after being confirmed as SARS-CoV-2 positive, and their nasopharyngeal swab (NPS), oropharyngeal swab (OPS), and sputum specimens were restored for SARS-CoV-2 retesting and respiratory pathogen detection. The majority of the subjects (96.5%, *N* = 330) were confirmed as SARS-CoV-2 positive using NPS/OPS specimens. Among the COVID-19 patients (*N* = 342), 7.9% (*N* = 27) and 0.9% (*N* = 3) were coinfected with respiratory viruses and *Mycoplasma pneumoniae*, respectively, yielding an 8.8% (*N* = 30) overall respiratory pathogen coinfection rate. Of the respiratory virus coinfection cases (*N* = 27), 92.6% (*N* = 25) were coinfected with a single respiratory virus and 7.4% (*N* = 2) with two viruses (metapneumovirus/adenovirus and rhinovirus/bocavirus). No triple coinfections of other respiratory viruses or bacteria with SARS-CoV-2 were detected. Respiratory viruses coinfected in the patients with COVID-19 were as follows: rhinovirus (*N* = 7, 2.1%), respiratory syncytial virus A and B (*N* = 6, 1.8%), non-SARS-CoV-2 coronaviruses (229E, NL63, and OC43, *N* = 5, 1.5%), metapneumovirus (*N* = 4, 1.2%), influenza A (*N* = 3, 0.9%), adenovirus (*N* = 3, 0.9%), and bocavirus (*N* = 1, 0.3%). In conclusion, the diagnostic value of utilizing NPS/OPS specimens is excellent, and, as the first report in Korea, coinfection with respiratory pathogens was detected at a rate of 8.8% in patients with COVID-19.

## 1. Introduction

COVID-19, caused by the infection with SARS-CoV-2, was identified from a cluster of pneumonia cases in Wuhan, China, in December 2019, and has spread to other countries since then, resulting in 42 million cases and more than 1.1 million deaths globally as of October 25, 2020 [1].

In Korea, a Chinese female from Wuhan, China, was identified as the first COVID-19 case on January 20, 2020, during a quarantine inspection at Incheon Airport [2]. The Korea government enforced SARS-CoV-2 testing not only for individuals with respiratory symptoms but also for those with histories of either international travel or contact with suspected cases, even when clinical symptoms were absent. Enforcement has caused exponential growth in real-time PCR (RT-PCR) tests to identify COVID-19 with emergency use authorized (EUA) *in vitro* diagnostic (IVD) assays [3].

For the rapid and accurate diagnosis of SARS-CoV-2 using IVD assays, selecting the appropriate types of specimens to be collected from patients at the right time is important [4]. Lower respiratory specimens such as bronchoalveolar lavage fluid and sputum have been recommended as the best clinical respiratory specimens for detecting SARS-CoV-2 [5, 6]. Viral loads of SARS-CoV-2 were higher in nasal swabs than those in throat swabs collected from symptomatic COVID-19 patients [7]. The United States Centers for Disease Control and Prevention has deemed upper respiratory specimens as acceptable for initial diagnostic testing of SARS-CoV-2, and these include nasopharyngeal swabs (NPS), oropharyngeal swabs (OPS), nasal swabs, and saliva [8]. Simultaneous collection and placement of NPS and OPS in the universal transport medium (UTM) using two sets of swabs is recommended to increase sensitivity in real-time polymerase chain reaction (RT-PCR) assays in Korea, as long as the supply of flocked swabs is not limited [9]. We analyzed the number of patients with COVID-19 that were confirmed as positive using NPS/OPS by measuring the detection rates of SARS-CoV-2 among the patients diagnosed during the screening process in the first outbreak of the disease in Korea.

The IVD assays for detecting SARS-CoV-2 were not incorporated for testing coinfections with other respiratory pathogens at the time of screening COVID-19 suspects. Recently, coinfections in COVID-19 have been reported not to neglect infections by other respiratory pathogens in addition to SARS-CoV-2 [10–14]. This suggests that simultaneous testing for coinfections between SARS-CoV-2 and other respiratory pathogens is required to provide a better patient treatment during the COVID-19 pandemic [15].

Among the respiratory pathogens, including *Mycobacterium tuberculosis*, viruses and bacteria have been the most commonly reported coinfection agents in COVID-19 patients, similar to those seen in the previous influenza pandemic [10, 12, 16, 17]. Viruses reported commonly in coinfection with SARS-CoV-2 included respiratory syncytial virus, influenza, rhinovirus/enterovirus, parainfluenza, metapneumovirus, and non-SARS-CoV-2 coronaviruses, and the coinfecting bacteria included *Mycoplasma pneumoniae*, *Legionella pneumophila*, *Chlamydia pneumoniae*, *Pseudomonas aeruginosa*, *Haemophilus influenzae*, and *Streptococcus pneumoniae* [10–13].

This study reports the simultaneous detection of SARS-CoV-2 and the coinfections with frequently reported respiratory viruses and bacteria causing atypical pneumonia (hereinafter atypical bacteria) using commercially available RT-PCR assays in upper (NPS and OPS) and lower (sputum) respiratory tract specimens collected from patients with confirmed cases of COVID-19.

## 2. Materials and Methods

### 2.1. Study Subjects

The request forms of those with suspected COVID-19 cases that were submitted to Seegene Medical Foundation, a nonprofit independent clinical reference laboratory, for SARS-CoV-2 testing from February 9 to 23, 2020, were retrospectively reviewed. The request forms were generated and recorded by government healthcare centers, hospitals, or quarantine offices with personal and clinical information, such as name, address, age, birth date, gender, specimen type and collection date, respiratory symptoms, and foreign travel history, based on the guidelines of the Korea Disease Control and Prevention Agency (KDCA). Once request forms with respiratory tract specimens (NPS/OPS and/or sputum) collected from the suspects were submitted, the test results were reported within 24 h of turnaround time. Based on the guidelines for testing COVID-19 in Korea, both upper respiratory tract samples (NPS and OPS) were placed in a UTM to increase test sensitivity [9, 18, 19].

We reviewed the request forms and enrolled study subjects who were confirmed as COVID-19 positive. The respiratory specimens (UTM containing NPS/OPS as upper respiratory specimens and sputum as the lower respiratory specimen) were restored from storage freezers (−70°C) and applied to extract nucleic acids to reconfirm SARS-CoV-2 and detect respiratory pathogens simultaneously.

### 2.2. Specimen Preparation and Nucleic Acid Extraction

The collected specimens were subjected to nucleic acid extraction as described below to detect SARS-CoV-2 and respiratory pathogens. A portion (200 *µ*L) of the UTM containing NPS/OPS was directly applied to the nucleic acid extraction. The sputum specimens were inspected for viscosity, homogenized, and diluted with phosphate-buffered saline as recommended by the specimen treatment guidelines of the Korean Society for Laboratory Medicine [9, 18]. Then, 200 *µ*L of diluted sputum was applied for extracting nucleic acids.

Nucleic acid extraction was performed by mixing the specimen (UTM or diluted sputum) and ready-to-use reagents from a commercial kit (MagNA Pure 96 DNA and Viral NA Small Volume Kit; Roche Applied Diagnostics, Mannheim, Germany) in MagNA Pure 96 instruments (Roche Diagnostics) according to the manufacturer's instructions. The final elution volume of the nucleic acids extracted from each specimen was approximately 100 *µ*L. The extracted nucleic acids were stored at −70°C and used to detect SARS-CoV-2 and respiratory pathogens.

### 2.3. Detection of SARS-CoV-2 and Respiratory Pathogens Using RT-PCR

To detect SARS-CoV-2, a commercial RT-PCR assay (Allplex^TM^ 2019-nCoV Assay, Seegene Inc., Seoul, Republic of Korea) was employed. In brief, the extracted nucleic acids (8 *µ*L) were mixed in a PCR tube containing One-step RT-PCR Mastermix (17 *µ*L), which consisted of Mono Oligo Mix (primers and probes; 5 *µ*L) to specifically detect three target genes of SARS-CoV-2 (envelope protein (E) gene, RNA-dependent RNA polymerase (RdRP) gene, and nucleocapsid protein (N) gene) [20], RNase-free water (5 *µ*L), Real-time One-step Buffer (5 µL), and Real-time One-step Enzyme (2 *µ*L). The mixing was carried out in an automated liquid-handling workstation (STARlet, Seegene Inc.) to maintain accuracy and prevent human errors. RT-PCR was performed in a CFX96 real-time PCR cycler (Bio-Rad, Hercules, CA, USA) as recommended by the manufacturer for detecting SARS-CoV-2.

The nucleic acids extracted from the specimens were also applied to simultaneously detect respiratory pathogens using commercially available multiplex real-time PCR assays (Allplex^TM^ Respiratory Panel 1, 2, and 3 assays for viruses and Allplex^TM^ PneumoBacter assay for bacteria, Seegene Inc.) as previously described [21]. The respiratory viruses detected using Allplex ^TM^ Respiratory Panel 1 were influenza A and B (Flu A and Flu B) and respiratory syncytial virus A and B (RSV). Those detected with Panel 2 were adenovirus (Adv), enterovirus (HEV), parainfluenza viruses 1, 2, 3, and 4 (PIV 1, 2, 3, and 4), and metapneumovirus (MPV), and those detected with Panel 3 were bocavirus 1/2/3/4 (HBoV), rhinovirus (HRV), and other coronaviruses (229E, NL63, and OC43). Allplex^TM^ PneumoBacter detected respiratory bacteria that cause atypical pneumonia (hereinafter atypical bacteria) such as *M*. *pneumoniae*, *C*. *pneumoniae*, *L*. *pneumophila*, and *Bordetella pertussis*.

The nucleic acids (8 *µ*L) extracted from specimens (UTM or diluted sputum) were also mixed in STARlet, as described above for detecting SARS-CoV-2, with the One-step RT-PCR Mastermix (17 *µ*L, consisting of primers, probes, Real-time One-step Enzyme, and buffer) from the commercial kits (Panel 1, 2, and 3 or PneumoBacter). RT-PCR was performed to simultaneously detect respiratory pathogens using CFX96 real-time PCR cyclers (Bio-Rad, Hercules, CA, USA) according to the manufacturer's instructions.

Interpretations for positive and negative detection of SARS-CoV-2 and respiratory pathogens by the multiplex RT-PCR assays were automatically determined using Seegene Viewer software provided by the manufacturer.

### 2.4. Statistical Analysis

The statistical significance of the differences in the detection rates of respiratory pathogens was analyzed using the chi-square or Fisher's exact tests. The mean value comparisons of age and Ct values among the study subjects were evaluated using the Mann–Whitney test. Statistical analysis was conducted using GraphPad Prism software (version 5.0; GraphPad Software, Inc., La Jolla, CA, USA) and GraphPad InStat software (version 3.0). *P* values < 0.05 were considered statistically significant.

### 2.5. Ethics Statement

We retrospectively analyzed the request forms and RT-PCR assay results of the study subjects, which were exempted from informed consent. This study was approved by the institutional review boards of Seegene Medical Foundation (SMF-IRB-2020-003) and Kyungpook National University Hospital (DGIRB202003001-HE002).

## 3. Results

### 3.1. Study Subject Enrollment

A total of 342 patients were enrolled in the study. After retrospectively reviewing the request forms and test results of suspects (*N* = 20,054) conducted for screening COVID-19 from February 7 to 23, 2020, we found that 404 patients (2.0%) were confirmed as SARS-CoV-2 positive based on the Allplex 2019-nCoV assay. Among those with positive test results, patients without personal information (*N* = 62; 15.3%), such as age, sex, and region of residence, were excluded. Finally, 342 subjects (84.7%) who were SARS-CoV-2 positive were enrolled.

The mean age ± SEM (standard error of the mean) of the study subjects was 47.8 ± 0.9 years (standard deviation: 15.7 years; 95% CI: 46.1–59.4 years) with a median age of 51.0 years. The detection rates of SARS-CoV-2 by the age were 0.9% (*N* = 3) for those below 20 years of age, 29.5% (*N* = 101) for those aged 20–39 years, 44.4% (*N* = 152) for those aged 40–59 years, and 25.1% (*N* = 86) for those aged > 60 years. This indicates that SARS-CoV-2 was mostly detected in those who were 40–59 years old.

The ratio of males to females was 37.1% (*N* = 127) and 52.9% (*N* = 215), respectively. The residential areas of the study subjects were predominantly Kyungpook Province (*N* = 320, 93.6%) including Daegu city, where the first outbreak of COVID-19 occurred in February, followed by Seoul (*N* = 8, 2.3%), Chungnam Province (*N* = 6, 1.8%), Kyunggi Province (*N* = 4, 1.2%), Kangwon Province (*N* = 3, 0.9%), and Jeonnam Province (*N* = 1, 0.3%).

### 3.2. Detection of SARS-CoV-2 by Types of Specimen

**Figure 1:**
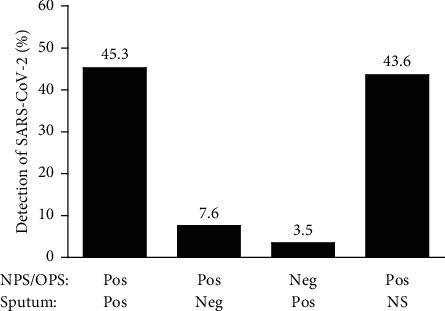
Classification of the study subjects by different types of respiratory specimens. Upper (NPS/OPS) and lower (sputum) respiratory specimens of the study subjects were retested for severe acute respiratory syndrome coronavirus 2 (SARS-CoV-2) using the Allplex 2019-nCoV assay. The study subjects (*N* = 342) were confirmed as positive for SARS-CoV-2 in both NPS/OPS and sputum at a rate of 45.3% (*N* = 155), SARS-CoV-2 positive in their NPS/OPS specimens but negative in their sputum at a 7.6% (*N* = 26) rate, and SARS-CoV-2 negative in their NPS/OPS specimens but positive in their sputum at a 3.5% (*N* = 12) rate. Some (43.6%, *N* = 149) were positive in NPS/OPS when sputum samples were not submitted. NPS: nasopharyngeal swab; OPS: oropharyngeal swab; NS: specimens were not submitted for COVID-19 (coronavirus disease 2019) testing; Pos: positive; Neg: negative.

Study subjects (*N* = 342) were retested for SARS-CoV-2 using the Allplex 2019-nCoV assay and classified as either positive for SARS-CoV-2 in both NPS/OPS and sputum (45.3%, *N* = 155), positive for SARS-CoV-2 in NPS/OPS but negative for SARS-CoV-2 in sputum (7.6%, *N* = 26), or negative for SARS-CoV-2 in NPS/OPS but positive for SARS-CoV-2 in sputum (3.5%, *N* = 12) (Figure 1). Some patients (43.6%, *N* = 149) were SARS-CoV-2 positive in NPS/OPS, although their sputum specimens were not submitted because they were not able to expectorate (Figure 1). Collectively, when the NPS/OPS specimens were tested only without accompanying sputum specimens, the positive rate of SARS-CoV-2 with the Allplex 2019-nCoV assay was 96.5% (*N* = 330) among the study subjects (*N* = 342). Among the study subjects (*N* = 193) for whom the sputum specimens were submitted and tested, the positive rate of SARS-CoV-2 was 86.5% (*N* = 167). These positive rates (95.5% and 86.5%) between testing NPS/OPS and the sputum specimen were significantly different (*P* < 0.0001). This suggests that NPS/OPS would be more valuable specimens than sputum for molecular diagnosis to detect SARS-CoV-2 (Figure 1).

**Figure 2:**
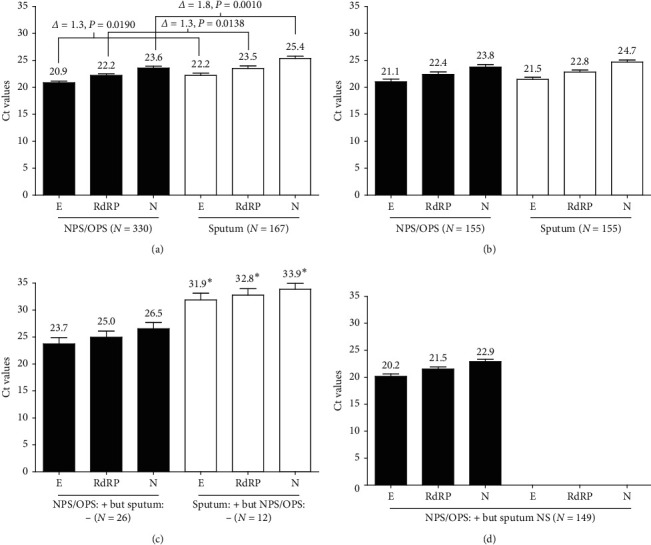
Ct values of the E, RdRP, and N genes in the Allplex 2019-nCoV assay for SARS-CoV-2 performed on nucleic acids extracted from the NPS/OPS and sputum specimens collected from the study group (*N* = 342). (a) Ct values of the E, RdRP, and N genes detected in NPS/OPS (*N* = 330) and sputum (*N* = 167) specimens among all study subjects (*N* = 342). (b) Ct values among the study subjects (*N* = 155) who were classified as positive for SARS-CoV-2 in both NPS/OPS and sputum samples. (c) Ct values among the study subjects (*N* = 26) who were classified as positive for SARS-CoV-2 in NPS/OPS but negative in sputum (NPS/OPS + but sputum −) and the subjects (*N* = 12) classified as negative for SARS-CoV-2 in NPS/OPS but positive in sputum (NPS/OPS – but sputum +). (d) Ct values among the study subjects (*N* = 149) who were classified as positive for SARS-CoV-2 in NPS/OPS even though their sputum specimens were not submitted. Ct: cycle threshold; SARS-CoV-2: severe acute respiratory syndrome coronavirus 2; NPS: nasopharyngeal swab; OPS: oropharyngeal swab; E: envelope protein gene; RdRP: RNA-dependent RNA polymerase gene; N: nucleocapsid protein gene.  ^*∗*^Significant difference; differences in Ct values of the E, RdRP, and N genes between those who were SARS-CoV-2 positive in tests on the NPS/OPS specimens but negative in tests on sputum (*N* = 26) and those who were negative in tests on their NPS/OPS specimens but positive in tests on their sputum (*N* = 12) were 8.2, 7.8, and 7.4, respectively, which were significantly different (*P* ≤ 0.0004).

The study subjects were confirmed as positive for SARS-CoV-2 when all three target genes in the Allplex 2019-nCoV assay were positive, as recommended by the Korea COVID-19 Diagnosis Testing Management Committee [18].  Figure 2 shows the mean cycle threshold (Ct) values of target genes for SARS-CoV-2 in the Allplex 2019-nCoV assay. The mean Ct values for NPS/OPS (*N* = 330) were 20.9 ± 0.3 (95% CI: 20.2–21.5) for the E gene, 22.2 ± 0.3 (95% CI: 21.6–22.8) for the RdRP gene, and 23.6 ± 0.3 (95% CI: 22.9–24.2) for the N gene. For sputum (*N* = 167), the mean Ct values for E, RdRP, and N genes were 22.2 ± 0.4 (95% CI: 21.3–23.1), 23.6 ± 0.4 (95% CI: 22.7–24.4), and 25.4 ± 0.4 (95% CI: 24.5–26.2), respectively. The differences (delta) of the mean Ct values for E, RdRP, and N genes between NPS/OPS and sputum were 1.3, 1.3, and 1.8, respectively. The mean Ct values for the E gene, RdRP gene, and N gene detected in NPS/OPS were significantly different from those in sputum (*P* ≤ 0.0138) (Figure 2(a)). The target genes of SARS-CoV-2 were detected significantly later in sputum than those in NPS/OPS when the Allplex 2019-nCoV assay was applied.

For the study subjects who were SARS-CoV-2 positive in both NPS/OPS and sputum (45.3%, *N* = 155) (Figure 1), the mean Ct values for the E, RdRP, and N genes in NPS/OPS were not significantly different (21.1 ± 0.5, 22.4 ± 0.5, and 23.8 ± 0.5, respectively) from those (21.5 ± 0.4, 22.8 ± 0.4, and 24.7 ± 0.4, respectively) in sputum (*P* ≥ 0.1249) (Figure 2(b)). However, as shown in Figure 2(c), the mean Ct values (23.7 ± 1.2, 25.0 ± 1.1, and 26.5 ± 1.1, respectively) for the E, RdRP, and N genes in the study subjects who were SARS-CoV-2 positive in NPS/OPS but negative in sputum (7.6%, *N* = 26) were significantly lower than those (31.9 ± 1.2, 32.8 ± 1.2, and 33.9 ± 1.1, respectively) who were SARS-CoV-2 positive in sputum but negative in NPS/OPS (3.5%, *N* = 12) (*P* ≤ 0.0004). The Ct values and viral load of SARS-CoV-2 are inversely proportional; therefore, the viral loads of SARS-CoV-2 in sputum were significantly lower than those in NPS/OPS. The viral loads of SARS-CoV-2 may influence the difference in the detection rates of the virus between NPS/OPS and sputum.

For the patients for whom NPS/OPS specimens were positive for SARS-CoV-2 when sputum was not submitted (43.6%, *N* = 149) (Figure 1), the mean Ct values (20.2 ± 0.4 for the E gene, 21.5 ± 0.4 for the RdRP gene, and 22.9 ± 0.4 for the N gene) (Figure 2(d)) were significantly lower than those for the patients who were SARS-CoV-2 positive with NPS/OPS but negative with the sputum specimen (Figure 2(c)) (*P* ≤ 0.0037), but statistically the same with those for the patients who were positive in both NPS/OPS and sputum specimens (Figure 2(b)) (*P* ≥ 0.1888).

### 3.3. Coinfection with Respiratory Pathogens in COVID-19

The coinfection rates with respiratory viruses and atypical bacteria among the study subjects (*N* = 342) were 7.9% (*N* = 27) and 0.9% (*N* = 3), respectively, which were significantly different (*P* < 0.0001) (Table 1). Among the respiratory pathogen coinfections (*N* = 30), 60.0% (*N* = 18) were detected in both NPS/OPS and sputum specimens, 26.7% (*N* = 8) only in NPS/OPS, and 13.3% (*N* = 4) only in sputum. Collectively, respiratory pathogen coinfections were detected with NPS/OPS at the rates of 86.7% (*N* = 26) and with sputum at 73.3% (*N* = 22) which were not significantly different (*P*=0.3334) (Table 1). It suggested that both types of specimens should be employed to detect coinfections with respiratory pathogens in patients with COVID-19.

Of the respiratory virus coinfection cases (*N* = 27), 25 of SARS-CoV-2-positive cases (92.6%) were coinfected with a single respiratory virus, and two cases (7.4%) (MPV/Adv and HRV/HBoV) were coinfected with two viruses. Respiratory viruses in single viral coinfection cases with SARS-CoV-2 were as follows: HRV (*N* = 6, 1.8%), RSV (*N* = 6, 1.8%; A (*N* = 2) and B (*N* = 4)), non-SARS-CoV-2 coronaviruses (*N* = 5, 1.5%; 229E (*N* = 3), NL63 (*N* = 1), and OC43 (*N* = 1)), MPV (*N* = 3, 0.9%), Flu A (*N* = 3, 0.9%), and Adv (*N* = 2, 0.6%).

All cases of respiratory bacterial coinfections (*N* = 3; male : female = 2 : 1) were *M*. *pneumoniae* detected in both NPS/OPS and sputum specimens without any other atypical bacteria detected (Table 1). Additionally, no triple coinfections of other respiratory viruses or bacteria with SARS-CoV-2 were detected.

The ratio of males to females in the coinfection cases was 4 : 6 (Table 1), but no difference in coinfection by gender was observed (*P*=0.3507), suggesting that both male and female confirmed as COVID-19 have the same level of susceptibility to coinfections with respiratory pathogens.

The mean age of the coinfection cases (*N* = 30, 8.8%) with respiratory pathogens (both viruses and bacteria) was 36.4 ± 2.9 years, which was significantly less than the mean age (48.6 ± 0.9) of patients without coinfections (*P* < 0.0001). The mean ages for the viral (*N* = 27) and bacteria (*N* = 3) coinfection cases were 36.9 ± 3.1 years and 32.0 ± 6.0 years, respectively, which were not statistically significant (*P*=0.6162) (Table 1).

Most of the respiratory pathogen coinfection cases were detected in Kyungpook Province (*N* = 27, 90%), followed by Kyunggi Province (*N* = 2 for each of Adv and HRV) and Seoul (*N* = 1 for HRV) (Table 1).

**Figure 3:**
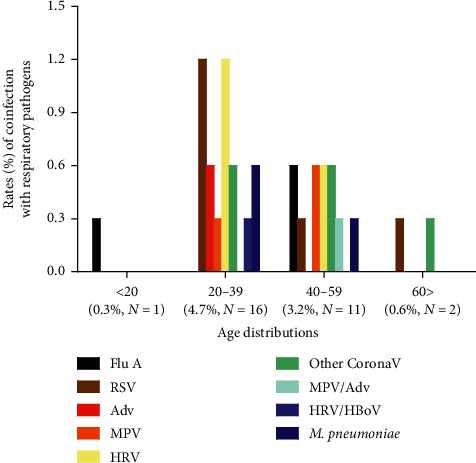
The distribution of respiratory pathogens by age groups in patients with COVID-19 and coinfections (*N* = 30) among the study subjects (*N* = 342). The number of coinfection cases based on the age distribution was 1 (0.3%), 16 (4.7%), 11 (3.2%), and 2 (0.6%) for ages < 20, 20–39, 40–59, and >60 years, respectively. COVID-19: coronavirus disease 2019; Flu A: influenza (A); RSV: respiratory syncytial virus; Adv: adenovirus; MPV: metapneumovirus; HRV: rhinovirus; Other CoronaV: coronaviruses such as 229E, NL63, and OC43; *M. pneumoniae*: *Mycoplasma pneumoniae*.

Figure 3 shows the age distribution of respiratory pathogen coinfection cases (*N* = 30) among the study subjects (*N* = 342). The rates of coinfections in the age groups of <20, 20–39, 40–59, and >60 years were 0.3% (*N* = 1), 4.7% (*N* = 16), 3.2% (*N* = 11), and 0.6% (*N* = 2), respectively. Among the coinfections with single respiratory virus in COVID-19 patients (*N* = 342), in the 20–39 age group, HRV (*N* = 4, 1.2%) and RSV (*N* = 4, 1.2%) were mostly detected, followed by Adv (*N* = 2, 0.6%), other coronaviruses (*N* = 2, 0.6%), and MPV (*N* = 1, 0.3%). In the 40–59 age group, 0.6% (*N* = 2) of Flu A, MPV, HRV, and other coronaviruses and 0.3% (*N* = 1) of RSV were coinfected. In the <20 age group, only Flu A (*N* = 1, 0.3%) was detected, and in the >60 age group, both RSV and other coronaviruses were detected in coinfections.

Two cases (0.6%) of double viral coinfections were found among those aged 20–39 (HRV/HBoV, *N* = 1, 0.3%) and 40–59 (MPV/Adv, *N* = 1, 0.3%), respectively. *M*. *pneumoniae* was detected as the coinfected atypical bacteria in those aged 20–39 (*N* = 2, 0.6%) and 40–59 (*N* = 1, 0.3%).

The occurrence of coinfections with respiratory pathogens in COVID-19 patients was not affected by viral loads of SARS-CoV-2. The mean Ct values of the target genes (21.2 ± 1.4 for the E gene, 22.4 ± 1.3 for the RdRP gene, and 23.8 ± 1.3 for the N gene in NPS/OPS and 22.9 ± 1.3 for the E gene, 24.2 ± 1.3 for the RdRP gene, and 25.9 ± 1.2 for the N gene in sputum) in the cases of coinfections (*N* = 30) with respiratory pathogens were not significantly different from those (20.8 ± 0.3 for the E gene, 22.2 ± 0.3 for the RdRP gene, and 23.6 ± 0.3 for the N gene in NPS/OPS and 22.1 ± 0.4 for the E gene, 23.4 ± 0.4 for the RdRP gene, and 25.3 ± 0.4 for the N gene in sputum) in COVID-19 cases without coinfections (*N* = 312) when the Allplex 2019-nCoV assay was employed, regardless of the types of respiratory specimen (*P* ≥ 0.5096).

## 4. Discussion

In Korea, both NPS and OPS were placed in a UTM after being collected from suspects of COVID-19 for screening [9]. The UTM was transported to the laboratory for COVID-19 testing immediately after collection, with or without sputum samples. No other upper or lower respiratory tract samples were collected and tested. Among the suspects (*N* = 20,054) tested from February 9 to 23, 2020, during the first outbreak in Korea, the positive rate of SARS-CoV-2 was 2.0% (*N* = 404) using an RT-PCR assay, which was lower than that found (9.5%; *N* = 116) in a previous study among patients tested in the USA (*N* = 1,217) between March 3 and 25, 2020 [22]. In this study, a total of 342 patients were enrolled among those who tested positive (*N* = 404) for SARS-CoV-2.

Among the study subjects (*N* = 342), 96.5% (*N* = 330) were positive for SARS-CoV-2 through the RT-PCR assay on NPS/OPS specimens, regardless of sputum collection (Figure 1). Additionally, based on a comparison of the mean Ct values for the genes specific to SARS-CoV-2, higher viral loads of SARS-CoV-2 were observed in NPS/OPS than in sputum (Figure 2), which supports that rates of SARS-CoV-2 detection in NPS/OPS are excellent (Figure 1). This suggests that NPS/OPS is better than sputum for detecting SARS-CoV-2 in molecular diagnostic assays and that it is unnecessary to enforce sputum sampling, especially from patients who are unable to expectorate. However, in previous studies, sputum specimens were considered superior to nasal samples for the molecular detection of SARS-CoV-2. Pan et al. [6] measured the viral loads of SARS-CoV-2 in the throats, sputum, urine, and stool of two COVID-19 patients at the early stage of symptom onset. They found that the viral loads were higher in sputum samples than in throat samples, especially in the early days after onset. Wang et al. [5] compared the detection rates of SARS-CoV-2 across multiple types of specimens collected from patients with COVID-19 and found that bronchoalveolar lavage fluid had the highest positive rate (93%), followed by sputum (72%), nasal swab (63%), and pharyngeal swabs (32%). Based on the literature published, bronchoalveolar lavage fluid was recommended as the best clinical specimen for detecting SARS-CoV-2 in patients, and sputum was the second most strongly recommended specimen, whereas pharyngeal swabs were given a moderate recommendation [23, 24]. In contrast, our data support that pharyngeal swab samples were superior to sputum for detecting SARS-CoV-2 among the study subjects who underwent COVID-19 screening. A finding consistent with that of the current study was also observed in a case study of two Korean patients, which reported that viral loads of upper respiratory specimens (placing both NPS and OPS in the same UTM) were similar to or sometimes higher than those in sputum using RT-PCR detection [25].

The rate of coinfection in patients with COVID-19 (*N* = 342) with respiratory pathogens was 8.8% (*N* = 30) (Table 1) and 7.9% (*N* = 27) and 0.9% (*N* = 3) having viral and atypical bacterial coinfections, respectively. The rate of viral coinfection has been previously reported to range from 2.0% to 19.8% in different countries [22, 26–28]. The coinfection rate with respiratory viruses was 2.0% (*N* = 39) among 1,996 COVID-19 patients hospitalized in New York City [26]. At a hospital in Shenzhen, China, six (3.2%) of 92 COVID-19 patients hospitalized were cases with viral coinfections [27]. Another study conducted in Wuhan, China, reported that, among patients with COVID-19 (*N* = 104), 5.8% (*N* = 6) had coinfections with non-SARS-CoV-2 coronaviruses (2.9%, *N* = 3), influenza A (2.9%, *N* = 3), and rhinovirus (1.9%, *N* = 2) [28]. In the study by Kim et al. [22] with patients in California, USA, 19.8% (*N* = 23) of SARS-CoV-2-positive patients (*N* = 116) were positive for other respiratory pathogens. As the first report on coinfections with respiratory pathogens among COVID-19 patients in Korea, especially in those from the first outbreak in February 2020, 7.9% were found to have viral coinfections, which is a rate comparable with those of previous reports.

Among the coinfections (*N* = 30) by respiratory viruses (*N* = 27) and the atypical bacterium *M*. *pneumoniae* (*N* = 3), most coinfection cases were detected from both NPS/OPS and sputum specimens (60.0%, *N* = 18), but some were detected only in either NPS/OPS (26.7%, *N* = 8) or sputum (13.3%, *N* = 4) (Table 1). This suggests that both upper and lower respiratory tract specimens should be used for monitoring coinfection by respiratory pathogens in COVID-19 patients. This is consistent with the conclusions of a previous report by Zhu et al. [14], who suggested that both upper and lower respiratory tract specimens should be collected for detecting respiratory pathogen coinfections in COVID-19 patients, and these specimens should be considered for testing during the diagnosis and treatment of COVID-19.

Based on a meta-analysis by Lansbury et al. [12] conducted with the previously published studies on the types of respiratory viruses detected as coinfection agents among patients with SARS-CoV-2, the most common coinfecting virus was RSV, followed by Flu A, HRV, PIV, and other coronaviruses. A study in China, which was not included in the meta-analysis, consistently reported that RSV was the most commonly detected among coinfecting viruses in COVID-19 patients diagnosed between January 19 and February 26, 2020 [15]. However, in the USA, Kim et al. [22] and Richardson et al. [26] detected rhinovirus/enterovirus as the most common coinfection agents, followed by RSV, among the study subjects they enrolled in March 2020. In this study with Korean study subjects enrolled in February 2020, the most common respiratory virus detected as a coinfection agent was HRV (2.1%, *N* = 7; one double viral coinfection with HBoV (HRV/HBoV) and 6 single viral coinfection cases in SARS-CoV-2-positive patients) (Table 1). Six cases (*N* = 1.8%) of RSV and 5 cases (*N* = 1.5%) of single viral coinfections by non-SARS-CoV-2 coronaviruses (three cases of 229E, one case of NL63, and one case of OC43) were also observed (Table 1).

SARS-CoV-2 and influenza coinfections were relatively rare and were observed in only three patients (0.9%) among the study subjects (*N* = 342) (Table 1). This finding is also consistent with those of previous studies, which documented rates of 0.54% in Turkey [13], 0.9% [22] and 2.4% [26] in the USA, and from 1.2% to 4.3% in China [14, 15, 28, 29].

Among the atypical bacteria tested, only *M*. *pneumoniae* was detected at the level of 0.9% (*N* = 3) of the study subjects (*N* = 342) (Table 1). In some other studies, however, *C*. *pneumoniae*, another atypical bacterium, was identified as another coinfection agent in patients with COVID-19 in addition to *M*. *pneumoniae* [14, 15, 26]. The coinfection rates in the above studies for *M*. *pneumoniae* and *C*. *pneumoniae* ranged from 1.6% to 4.8% and 2.5%–5.2%, respectively. Lansbury et al. [12] emphasized, in their meta-analysis study, that *M*. *pneumoniae* was the most common bacteria detected in patients with COVID-19 who had respiratory bacterial coinfections, followed by *P*. *aeruginosa*, *H*. *influenza*, *Klebsiella pneumoniae*, and *Chlamydophila* spp., suggesting that in addition to coinfection by atypical bacteria, other respiratory bacteria were also the candidates of coinfections in COVID-19 patients.

Unlike Kim et al. [22] who reported no difference in age between coinfection and SARS-CoV-2 only (non-coinfection) groups, the mean ages of these groups were significantly different in this study (36.4 ± 2.9 vs. 48.6 ± 0.9 years old, respectively; *P* < 0.0001), where most coinfections with respiratory pathogens were detected at ages between 20 and 39 (*N* = 16, 4.7%) (Figure 3). This suggests that young individuals with COVID-19 are more susceptible to respiratory pathogen coinfections.

The viral loads of SARS-CoV-2 in COVID-19 patients with coinfection and patients without coinfections were not significantly different as Ct values for the target genes (E, RdRP, and N) were statistically the same between these patient groups when the Allplex 2019-nCoV assay was applied (*P* ≥ 0.5096). This suggests that the viral load of SARS-CoV-2 is not responsible for causing respiratory pathogen coinfections.

The current study had several limitations. First, the effects of coinfections on the treatment outcomes of COVID-19 patients are unknown. The study subjects were retrospectively enrolled during the COVID-19 screening process in the region of the first outbreak in Korea. Thus, the scope of this study was to investigate the coinfection rates of respiratory pathogens among patients with COVID-19 who were not hospitalized, but diagnosed as positive for SARS-CoV-2 during the screening process. Second, the results of the study cannot represent the coinfection rates in winter when respiratory pathogens such as influenza are dominant. The study subjects were enrolled only in February; therefore, it would be interesting to investigate the rates of coinfection with respiratory pathogens across the entire winter season, especially from November to March. Third, the commercial multiplex RT-PCR assays (Allplex^TM^ Respiratory Panel 1, 2, and 3) that we employed were able to detect the subtypes found in Flu, RSV, PIV, and non-SARS-CoV-2 coronaviruses as described in Materials and Methods but unable to distinguish the HRV subtypes (A, B, and C), which were previously included in a report by Kuypers et al. [30]. Finally, this study did not investigate coinfections in COVID-19 patients with either opportunistic pathogens such as *Streptococcus* and *Actinomyces* or fungi such as *Aspergillus* and *Candida*, as Chen et al. [10, 31] described. The broad range of coinfecting agents in COVID-19 patients should be investigated.

## 5. Conclusions

In summary, the upper respiratory tract specimens (NPS/UPS) provided excellent detection of SARS-CoV-2 without lower respiratory specimens (sputum) in the RT-PCR assays. However, the detection of coinfections with respiratory pathogens in COVID-19 patients requires both upper and lower respiratory specimens. The rate of respiratory pathogen coinfections in patients with COVID-19 (*N* = 342) was 8.8% (*N* = 30), with most of the coinfecting agents being viruses (7.9%, *N* = 27) and an atypical bacterium, *M*. *pneumoniae* (0.9%, *N* = 3). The most common respiratory viruses detected as coinfecting agents were HRV (*N* = 7, 2.1%), followed by RSV (*N* = 6, 1.8%), non-SARS-CoV-2 coronaviruses (*N* = 5, 1.5%), MPV (*N* = 4, 1.2%), Flu A (*N* = 3, 0.9%), Adv (*N* = 3, 0.9%), and HBoV (*N* = 1, 0.3%). In conclusion, simultaneous detection of respiratory pathogens and SARS-CoV-2 by molecular diagnosis assays is necessary for identifying the causative agents of coinfection, especially during the COVID-19 pandemic.

## Figures and Tables

**Table 1:** Study subjects (*N* = 342) with COVID-19 coinfected with respiratory pathogens.

Patients coinfected with respiratory pathogens		No. of patients coinfected (%)	M : F	Mean age (SEM)	Region of residency (no. of patients)	Classification of the coinfections by types of specimen (*N* = 25)		
						No. (%) of SARS-CoV-2 positive in		
						NPS/OPS	Sputum	Both
Virus (*N* = 27, 7.9%)	Flu A	3 (0.9)	1 : 2	30.3 (14.7)	KP (3)	2 ^*∗*^	—	1
	RSV	6 (1.8)	1 : 5	37.5 (8.3)	KP (6)	2 ^*∗*^	2	2
	Adv	2 (0.6)	2 : 0	29.0 (8.0)	KP (1),	—	1	1
					KG (1)			
	MPV	3 (0.9)	1 : 2	40.3 (8.0)	KP (3)	—	—	3
	HRV	6 (1.8)	2 : 4	37.2 (6.4)	KP (4),	3 ^*∗*^	—	3
					Seoul (1)			
					KG (1)			
	Non-SARS-CoV-2 coronavirus	5 (1.5)	3 : 2	39.8 (6.9)	KP (5)	—	1	4
	MPV/Adv	1 (0.3)	0 : 1	53	KP (1)	1	—	—
	HRV/HBoV	1 (0.3)	0 : 1	25	KP (1)	—	—	1

Bacteria (*N* = 3, 0.9%)	*M*. *pneumoniae*	3 (0.9)	2 : 1	32.0 (6.0)	KP (3)	—	—	3
Total (*N* = 30, 8.8%)		30	12 : 18	36.4 (2.9)		8 (26.7)	4 (13.3)	18 (60.0)

COVID-19: coronavirus disease 2019; Flu A: influenza A; RSV: respiratory syncytial virus; Adv: adenovirus; MPV: metapneumovirus; HRV: rhinovirus; Non-SARS-CoV-2 coronavirus: coronaviruses 229E, NL63, and OC43; *M*. *pneumoniae*: *Mycoplasma pneumoniae*; KP: Kyungpook Province; KG: Kyunggi Province; SEM: standard error of mean. ^*∗*^NPS/OPS specimens were submitted only without sputum.

## Data Availability

The data used to support the findings of this study are available from the corresponding author (Dr. Nackmoon Sung (paratb@gmail.com)) upon request. Requests will be reviewed by coauthors and decided to provide.
